# So many, yet few: Human resources for health in India

**DOI:** 10.1186/1478-4491-10-19

**Published:** 2012-08-13

**Authors:** Krishna D Rao, Aarushi Bhatnagar, Peter Berman

**Affiliations:** 1 Public Health Foundation of India, New Delhi, India; 2 Johns Hopkins Bloomberg School of Public Health, Baltimore, MD, USA; 3 Harvard School of Public Health, Boston, MA, USA

**Keywords:** India, Human resources, Census, Household survey

## Abstract

**Background:**

In many developing countries, such as India, information on human resources in the health sector is incomplete and unreliable. This prevents effective workforce planning and management. This paper aims to address this deficit by producing a more complete picture of India’s health workforce.

**Methods:**

Both the Census of India and nationally representative household surveys collect data on self-reported occupations. A representative sample drawn from the 2001 census was used to estimate key workforce indicators. Nationally representative household survey data and official estimates were used to compare and supplement census results.

**Results:**

India faces a substantial overall deficit of health workers; the density of doctors, nurses and midwifes is a quarter of the 2.3/1000 population World Health Organization benchmark. Importantly, a substantial portion of the doctors (37%), particularly in rural areas (63%) appears to be unqualified. The workforce is composed of at least as many doctors as nurses making for an inefficient skill-mix. Women comprise only one-third of the workforce. Most workers are located in urban areas and in the private sector. States with poorer health and service use outcomes have a lower health worker density.

**Conclusions:**

Among the important human resources challenges that India faces is increasing the presence of qualified health workers in underserved areas and a more efficient skill mix. An important first step is to ensure the availability of reliable and comprehensive workforce information through live workforce registers.

## Background

Greater availability of health workers is associated with better service utilization and health outcomes [1-3]. In addition to overall numerical strength, health workforce effectiveness is also influenced, among other things, by skill mix, type of providers and their geographical distribution. Information on indicators such as these is critical for policy makers to manage and plan better for the health workforce. Yet, in many developing countries, such as India, workforce planning is handicapped by the lack of comprehensive and reliable information on the number of health workers, what types operate, what their qualifications are and where they are located.

Counting health workers in India is a challenging exercise. For one, India’s health workforce is characterized by a diversity of health workers offering health services in several systems of medicine. These health workers are present in both the private and public sector. According to the National Occupation Classification (NOC), providers of allopathic health services broadly include doctors (general and specialists), dentists, nurses, midwives, pharmacists, technicians, optometrists, physiotherapists, nutritionists, sanitarians and a range of administrative and support staff [4]. Physicians and surgeons trained in Indian systems of medicine - Ayurveda, Yoga, Unani, Sidha - and Homeopathy, collectively known as AYUSH, also provide health care through public and private sector facilities. Certain states have also introduced state specific cadres; the states of Chhattisgarh and Assam have deployed non-physician clinicians with three and a half years of allopathic training. In addition, a large number of community health workers operate in the health sector.

Adding to this complexity is the large number of informal medical practitioners, commonly called RMPs (Registered Medical Practitioners ^a^). RMPs are often the first point of contact for medical care for the rural population and the urban poor. They typically practice allopathic medicine, but have no formal qualification or license to do so. While it is difficult to estimate their numbers, one study estimates that 25% (42% in rural and 15% in urban) of the individuals classified as allopathic doctors, reported no medical training [5]. Another study conducted in the Udaipur district of Rajasthan in 2003 found that 41% of private practitioners who called themselves doctors had no medical degree, 18% had no medical training at all and 17% had not even graduated from high school [6]. In addition, a substantial number of practitioners of traditional medicine and faith healers inhabit the rural workforce space.

Routine sources of information on the health workforce are fragmented and generally unreliable. For certain cadres (allopathic doctors, AYUSH physician, dentists, nurses, pharmacists) of health workers, information on their strength is available from their respective professional councils. However, this information suffers from several limitations. Because professional councils don’t maintain live registers, the information they provide is inaccurate due to non-adjustment of health workers leaving the workforce due to death, migration and retirement or double counting of workers due to their registration in more than one state [7]. Further, not all state councils follow the same registering procedure, raising issues of comparability. Importantly, certain categories of health workers, such as physiotherapists, medical technicians, RMPs and faith healers, are not recorded at all. Finally, data on health workers in some states (e.g. India’s north-east) are not available because they do not have state specific professional councils.

This paper attempts to present a more complete picture of India’s health workforce. It quantifies the size, composition and distribution of India’s health workforce by drawing on non-routine sources such as the Census and from nationally representative household surveys. Because these sources collect information directly from individuals, they can potentially overcome many of the deficiencies associated with routine data sources.

## Data and methods

This study used data from two sources - the 2001 Census of India and the 61st round (July 2004-June 2005) of the National Sample Survey (NSS) on ‘Employment and Unemployment’. The census data were a sample drawn from the population - from each district of the country, 20% of the rural and 50% of the urban enumeration blocks (EB) were selected using systematic sampling. An EB consisted of 600 and 750 individuals in the urban and rural areas, respectively. In the 11 smaller states and union territories (<2 million population) all EBs were selected, making the total sample size roughly 300 million individuals. The sample estimates were then inflated by a factor of five for rural and two for urban districts to get population totals.

The NSS is a multi-stage stratified cluster sample survey covering the entire country. This survey was spread over 7999 villages and 4602 urban blocks covering 124 680 households and 602 833 persons. Both the census and the NSS collected information on the self-reported occupation [8].

The National Occupational Classification (NOC) codes were used to classify occupation self-reports [4]. NOC codes enabled classifying health workers according to their specific occupation such as doctors, nurses, homeopaths, ayurvedic practitioners, medical assistants, traditional and faith healers and the like. These were grouped and the final categories of health workers included allopathic physicians, AYUSH practitioners, nurses and midwives, dentists, pharmacists, others (including the paramedical support staff) and other practitioners of traditional medicine [9]. The category of nurses and midwifes was grouped together as their NOC codes suggested overlapping job functions. Similarly, it is possible that traditional birth attendants are subsumed under midwifes because the NOC codes do not distinguish between the two.

Because workforce information from the Census and the NSS is based on occupation self-reports, it is susceptible to unqualified providers being counted as qualified ones. To adjust for this, data from the NSS, which collected information on both occupation and technical education (degree or diploma/certificate in medicine) and general education, was used to calculate the proportion of qualified health workers and this fraction was then applied to the Census estimates. For instance, a person classified as an allopathic doctor was considered qualified if they either had a technical degree or post-graduate diploma/certificate in medicine. Persons classified as nurses and midwives were considered qualified if they had any technical education in medicine or if they possessed a diploma/certificate.

To make the Census and NSS estimates temporally comparable, the average annual population growth rate between 1991 and 2001 Census was used to upwardly adjust the 2001 Census estimates to 2005.

## Results

### Size and composition^b^

**Figure 1:**
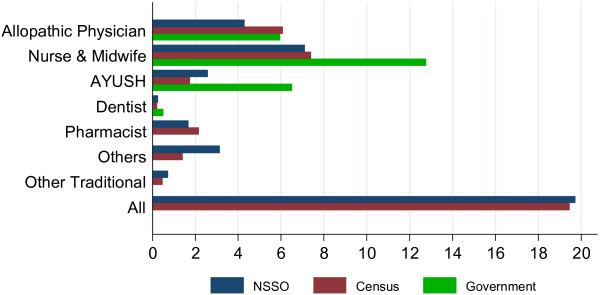
Health worker density - All India (Per 10 000 population).

The Census estimates show that there were approximately 2.17 million health workers in India in 2005, which translates into a density of approximately 20 health workers per 10 000 population (Figure 1). Among the different categories of health workers shown in Figure 1, nurses and midwifes had the largest share in the health workforce, followed by allopathic physicians, AYUSH physicians and pharmacists. The Census and NSS estimates are remarkably close in the estimated total number of health workers although there are differences when the data are broken down by cadres. Government estimates of workers in both the public and private sector are only available for some cadres. In general, across cadres, the Census and NSS estimates tend to be closer to each other than the Government estimates.

**Figure 2:**
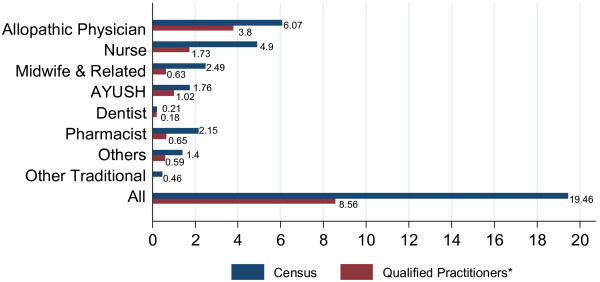
Health worker density - All India, 2005 (Per 10 000 Population).

When the Census estimates are adjusted for health worker qualification the health worker density reduced from 20 to a little over 8 per 10 000 population (Figure 2). For physicians, estimates from the NSS survey suggest that 37% (63% in rural and 20% in urban areas) had inadequate or no medical training; applying this proportion to the Census estimates, the allopathic physician density in India reduced from 6.1 to 3.8 per 10 000 population. In rural (urban) areas the qualified allopathic physician density is 1.2 (11.3) per 10 000 population. Put another way, there is one qualified doctor per 8333 (885) people in rural (urban) areas of India.

There are 4.9 nurses and 2.5 midwifes per 10 000 population. This translates to 1.6 nurses and midwifes per allopathic physician. After adjusting for unqualified workers, the nurse density reduces to 1.7 and the midwife to 0.6 per 10 000 population making the nurse-doctor ratio as low as 0.5.

### Distribution

**Figure 3:**
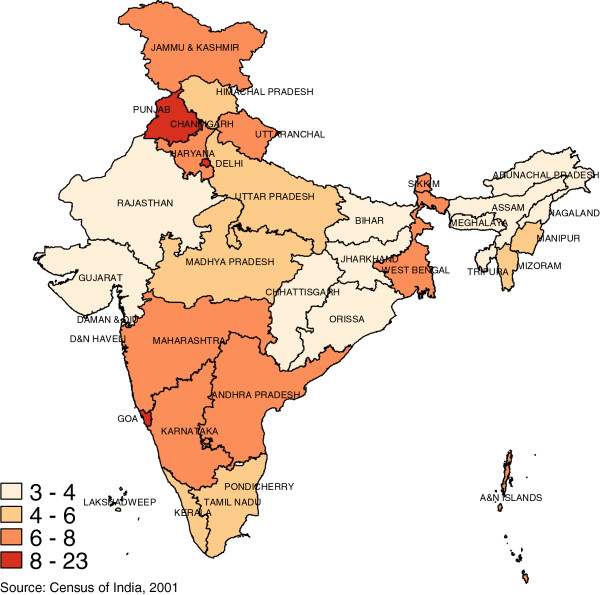
Doctor density, 2005 (Per 10 000 Population).

**Figure 4:**
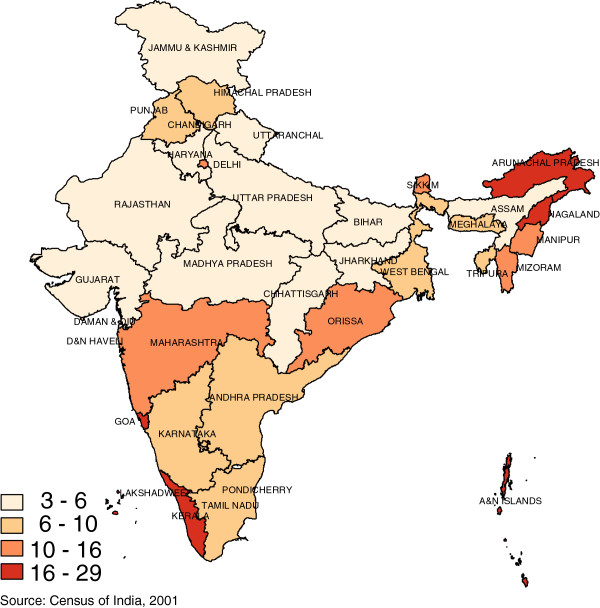
Nurse & Midwife density, 2005 (Per 10 000 Population).

There is considerable variation in the density of the health workforce across the states of India. For example, Figure 3 shows that states such as Goa and Kerala have doctor densities up to three times as high as states such as Orissa and Chhattisgarh. Similarly, variation in nurse and midwife density (Figure 4) in states such as Goa and Kerala are up to six times as much as the low density states of Bihar and Uttar Pradesh. In general, the north-central states have low workforce densities and also have poorer average health.

**Figure 5:**
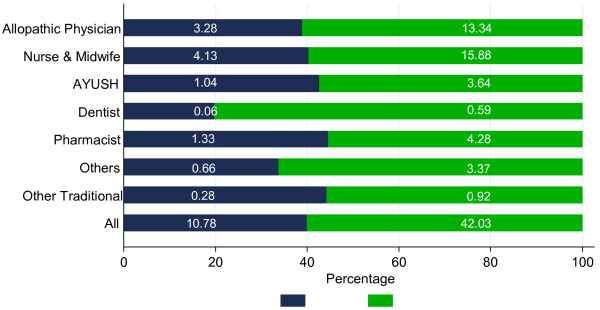
Rural–urban distribution of health workers in India, 2005.

The majority (60%) of health workers are present in urban areas (Figure 5). Because the majority of India’s population is rural, health worker to population ratios are even more skewed. For example, the density of allopathic physicians in urban areas is four times that of rural areas, and for nurses and midwives it is three times as large. If the NSS estimate of the proportion of unqualified allopathic physicians were applied, then the density of allopathic physicians in urban and rural areas would be 11.3 and 1.2, respectively, reflecting the higher proportion of physicians reporting insufficient qualifications in rural areas. Similarly, the density of qualified nurses is higher in urban (4.3) relative to rural (0.7) areas.

**Figure 6:**
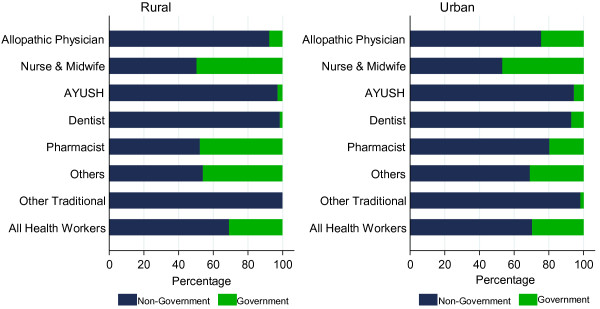
Distribution of health workforce by sector, 2005.

The majority (70%) of health workers were employed in the private sector in both urban and rural areas (Figure 6). Significantly, the vast majority of doctors, AYUSH practitioners and dentists were employed by the private sector in both urban and rural areas. In contrast, only about half the nurses were employed by the private sector. Health workers without qualifications were mainly present in the private sector.

**Figure 7:**
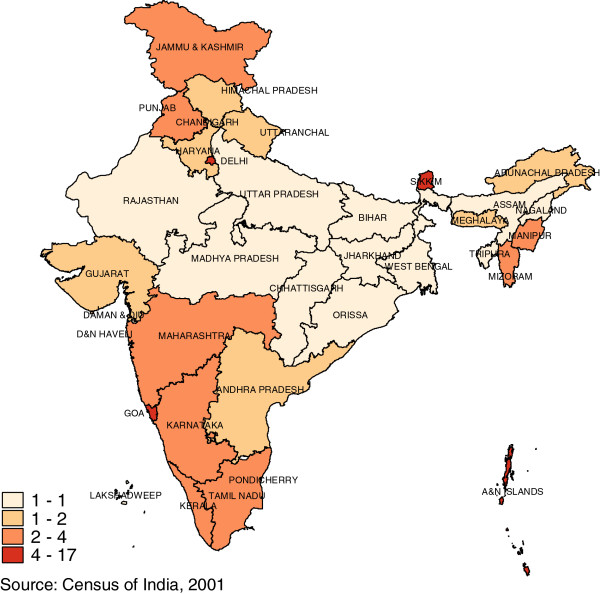
Female doctor density, 2005.

The proportion of women in the health workforce is low. There are approximately 7 female health workers per 10 000 population, indicating that women comprise only about a third of all health workers in the country. There were only about 2 female doctors per 10 000 women in the population. The share of female doctors was particularly low comprising only 17% of all doctors in the country (Figure 7) and only 6% of the rural doctors. In contrast, 70% of nurses and midwives were women.

Health workforce estimates presented here do not include community workers, although these are intended in part to address the low access to more qualified workers. The Census and NSS, which classify health workers based on international occupation codes, do not have separate classification codes for community health workers. At the time of the 2001 Census and the 2004/2005 NSS, Accredited Social Health Activists’ (ASHA) were not yet introduced into the workforce. Under the National Rural Health Mission (NRHM) the Government will add more than five hundred thousand ASHAs to the health workforce [10]. Further, nearly one million community workers for the Integrated Child Development Scheme [11] are also not included in the health workforce estimates. Both these groups of health workers would add a significant number to the health workforce, especially in rural areas. The inclusion of community workers would increase the size of the health workforce in India by nearly 80%.

### Workforce density and health

**Figure 8:**
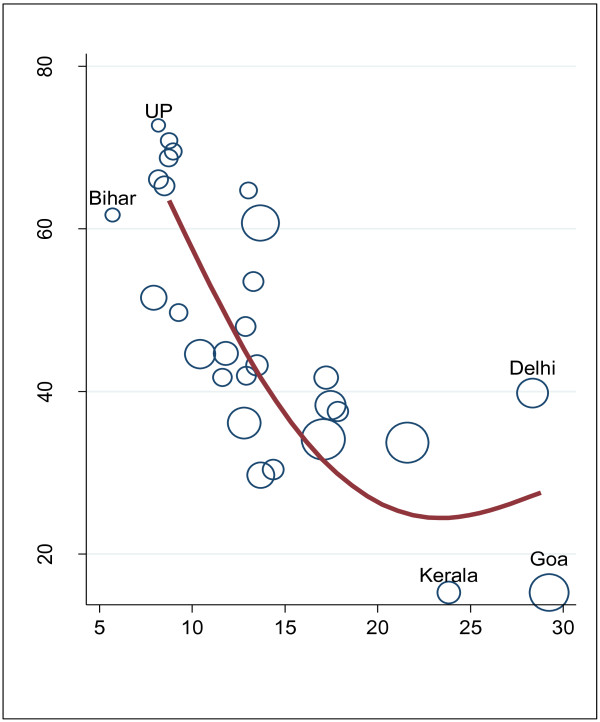
Workforce density and infant mortality.

States with higher health worker density tend to have lower infant mortality rates and better health, more generally (Figure 8). Similarly, positive associations are observed for immunizations and attended deliveries (results not shown). Bihar and Uttar Pradesh have low health worker density and poor health, while Goa and Kerala are at the opposite extreme. Interestingly, there is considerable variation in infant mortality for given density levels indicating that there are several factors other than workforce availability which influence health and service utilization. It also suggests that some states have more efficient health workers.

Higher per capita state spending on health, workforce density and health appear to be associated. In general, states with higher per capita health spending have higher workforce density and better health outcomes. Again, Goa with higher government spending on health has a higher health worker density and substantially lower infant mortality compared to states such as Bihar and Uttar Pradesh. This is expected since the majority of state health spending is on workforce salaries.

## Discussion and conclusion

In many developing countries such as India, policy makers lack basic information on the health workforce which handicaps effective planning and management. Building a reliable and comprehensive information system will require fundamental changes in the scope and manner in which workforce data are collected. Some of these changes are relatively easy to implement; for example, maintaining live registers for different cadres of health workers. Other measures such as registering unqualified health workers are more challenging but vital to be able to better regulate health providers. The level at which workforce information is collected is also important. Current routine sources of workforce information are typically available only at the state level. Disaggregating this information to the district level will make it considerably more useful for resource management for several reasons. India has large districts with considerable variation in population and geography between districts within states. Further, health systems planning is now done upwards from the district level which makes it important to have reliable information on health workers in a district.

Information contained in non-routine information sources can provide a rich and comprehensive description of the health workforce. This study illustrates the use of the Census and household surveys for this purpose. Comparisons between the NSS and Census indicate that the latter has good validity. Because of the opaque way in which professional councils in India count health workers it is not possible to say anything about the validity of officially reported health workforce estimates.

The Census results paint a dismal picture of the health workforce landscape. For one, there is an overall deficit in the number of qualified health workers; the estimated density of allopathic physicians, nurses and midwifes (13.4) in 2005 was about half of the WHO benchmark of 22.8 workers of these categories per 10 000 population associated with achieving 80% deliveries attended by skilled personnel in cross-country comparisons [12]. When adjusted for possible inclusion of unqualified providers, the level may be as low as one fourth of the WHO benchmark. This highlights both the deficit of qualified health workers in India’s health sector as well as the large number of unqualified health workers operating in the workforce, particularly in rural and poor urban areas.

The geographic mal-distribution of the health workforce in India is another cause for concern. States with poor health indicators tend to have fewer health workers. While several factors drive health outcomes, having few health workers profoundly influences the ability of the health systems to deliver preventive and curative services. The large disparity in workforce density between urban and rural areas is alarming. This rural shortage is due to a lack of qualified health workers in both the public and private sector. The rural deficit indicates the difficulty rural Indians face in accessing health care from qualified health workers and their reliance on unqualified providers. Further, efforts to increase the coverage and quality of health services in rural areas are also severely constrained by the lack of qualified health workers thereby providing lucrative opportunities for unqualified providers to fill this need. This is further compounded by a lack of regulation provided by the government and professional bodies which play a poor role in regulating even qualified health workers [13].

The reasons behind the geographic mal-distribution of qualified health workers need to be better understood through focused research on the supply side (e.g., production capacity of health workers) and the demand side (e.g., incentives to recruit and retain, institutional factors and policy environment) factors [14-17]. The large urban bias in the distribution of qualified health workers can be addressed by changing the incentive environment in which health workers operate. For this, a better understanding of the effectiveness of, and experimentation with, different strategies to attract and retain health workers in rural areas is necessary. Several of these experiments are currently underway in different states in India and these should be closely watched; they represent local solutions to a national problem.

Findings from this study also draw attention to the sub-optimal mix of health workers in the workforce - the nurse-doctor ratio in India is heavily skewed in favour of doctors. Having similar number of nurses and physicians is widely seen internationally as a significant imbalance in the human resource skill mix. In comparison, countries like the United States of America and the United Kingdom have nurse-physician ratios of 3 and 5, respectively [1]. According to the 1993 World Development Report, as a rule of thumb, the ratio of nurses to doctors should exceed 2:1 as a minimum with 4:1 or higher considered more satisfactory for cost-effective and quality care [18]. The limited presence of nurses in India’s health workforce is a reflection of the poor representation of female health workers, particularly doctors, in the workforce. This underrepresentation of women indicates forgone opportunities for women to participate in the health workforce and will likely have an effect on the uptake of maternal health services, particularly in rural areas.

Nurses and other mid-level cadres of health workers can deliver many of the basic clinical and public health services, particularly at the community level, at a lower cost than trained physicians. Further, such cadres are likely to be more amenable to join government service, as nurses in India are (see Figure 6), and more easily placed in underserved areas. Already in two states (Chhattisgarh and Assam), non-physician clinicians have been deployed to address the rural health worker deficit. The use of such cadres to deliver certain basic clinical services offers a way of reducing the substantial doctor deficit in rural India.

The estimates derived from the Census closely match those from the NSS, thereby suggesting that the Census estimates have good validity. However, the accuracy of workforce information from non-routine sources such as the Census and household surveys can be improved in several ways. For one, information on self-reported occupations should be crosschecked with the reported educational qualifications. This helps in separating out qualified and less qualified health workers and produces more reliable estimates for both. Secondly, the current classification codes used in the census are not sensitive enough to detect some health worker cadres such as community health workers, traditional birth attendants and community based nutrition workers. With India investing in these types of health workers in a major way, enumerating them is all the more important.

## Endnotes

^a^ The term RMP comes from the registration decades ago of non-physician providers with limited or in some cases no qualifications. Despite changes in the regulations, today most RMPs are not “registered” nor recognized, yet the term persists.

^b^ Estimates presented in this section do not distinguish between qualified and unqualified health workers, unless specifically stated.

## Competing interests

The authors declare that they have no competing interests.

## Authors’ contributions

KDR and AB were primarily responsible for writing the manuscript and data analysis. PB contributed to conceptualizing the study, manuscript writing and provided guidance. All authors read and approved the final manuscript.
